# Re-Identification of Patient Subgroups in Uveal Melanoma

**DOI:** 10.3389/fonc.2021.731548

**Published:** 2021-10-20

**Authors:** Thi Hai Yen Nguyen, Tin Nguyen, Quang-Huy Nguyen, Duc-Hau Le

**Affiliations:** ^1^ Department of Computational Biomedicine, Vingroup Big Data Institute, Hanoi, Vietnam; ^2^ Department of Computer Science and Engineering, University of Nevada, Reno, Reno, NV, United States; ^3^ College of Engineering and Computer Science, VinUniversity, Hanoi, Vietnam

**Keywords:** uveal melanoma, clustering, multi-omics, molecular subtypes, biomolecular markers

## Abstract

Uveal melanoma (UM) is a comparatively rare cancer but requires serious consideration since patients with developing metastatic UM survive only for about 6–12 months. Fortunately, increasingly large multi-omics databases allow us to further understand cancer initiation and development. Moreover, previous studies have observed that associations between copy number aberrations (CNA) or methylation (MET) versus messenger RNA (mRNA) expression have affected these processes. From that, we decide to explore the effect of these associations on a case study of UM. Also, the current subtypes of UM display its weak association with biological phenotypes and its lack of therapy suggestions. Therefore, the re-identification of molecular subtypes is a pressing need. In this study, we recruit three omics profiles, including CNA, MET, and mRNA, in a UM cohort from The Cancer Genome Atlas (TCGA). Firstly, we identify two sets of genes, CNAexp and METexp, whose CNA and MET significantly correlated with their corresponding mRNA, respectively. Then, single and integrative analyses of the three data types are performed using the PINSPlus tool. As a result, we discover two novel integrative subgroups, IntSub1 and IntSub2, which could be a useful alternative classification for UM patients in the future. To further explore molecular events behind each subgroup, we identify their subgroup-specific genes computationally. Accordingly, the highest expressed genes among IntSub1-specific genes are mostly enriched with immune-related processes. On the other hand, IntSub2-specific genes are highly associated with cellular cation homeostasis, which responds effectively to chemotherapy using ion channel inhibitor drugs. In addition, we detect that the two integrative subgroups show different age-related risks and survival rates. These discoveries can influence the frequency of metastatic surveillance and support medical practitioners to choose an appropriate treatment regime.

## 1 Introduction

Uveal melanoma (UM) is a comparatively rare cancer formed from melanocytes within the uveal tract of the eye involving either in the iris, ciliary body, or mostly choroid (1) and responsible for about five cases per million per year (2). Although current first-line treatment approaches receive good results for this malignancy, specifically, UM patients can live longer, but we want to improve early diagnosis more with the hope of raising overall patient survival as smaller tumors are treated, resulting in achieving local disease control and vision preservation with the possibility to prevent metastases (3). However, it has still remained challenging. Indeed, UM patients with the metastatic disease only lived for approximately 6–12 months (4). This emphasizes a pressing need of improving the diagnosis, prevention, and treatment of UM patients.

Besides, several recent large-scale and multi-omics databases have enabled us to see associations between the genetic or epigenetic alterations versus the tumorigenesis and progression of UM. For example, the importance of different types of RNA such as mRNA, microRNA (miRNA), and long non-coding RNA (lnCRNA) was investigated in UM (5, 6). Based on an *in silico* and experimental biology, lnCRNA LINC00518 was identified to be a oncogene in UM and could be used in RNA-based therapeutic approaches as a promising target (6). Additionally, UM has frequently had copy number aberrations (CNA) gain regions of chromosomes 6p and 8q as well as loss regions of chromosomes 1p, 3, 6q, 8p, and 16q (7, 8). Particularly, *BAP1* mutations related to chromosome 3 monosomy and *SF3B1* and *SRSF2* alterations related to chromosome 3 disomy contributed to high risk of metastasis. Meanwhile, mutations on *EIF1AX* related to chromosome 3 disomy were associated with low metastatic risk (9). In addition, Yang et al. (4) have made a comprehensive review of the role of DNA methylation in the development and metastasis of UM. They highlighted that several tumor suppressor genes comprising *RASSF1A* and *p16INK4a* have been altered by DNA methylation (MET) and contributed to controlling cell migration and invasion in UM. Moreover, *p16INK4a* expression was reported in all UM liver metastatic cases and may have potential in discriminating UM and cutaneous melanoma (10). Besides, the autophagy has been hypothesized to have a role in inhibiting tumor growth when investigating this process-related protein, Beclin-1. The high level of immunohistochemistry in Beclin-1 was found to be a positive prognosis of UM patients (11).

Moreover, multiple prior studies have been conducted to stratify UM patients using various kinds of -omic data. Among them, the most popular work proposed by Robertson et al. (5) has conducted a multiplatform analysis of 80 UM patients using only one single data type of omics data, including mRNA expression, miRNA, long non-coding RNA, MET, and CNA, and successfully identified four different subtypes: two associated with poor-prognosis monosomy 3 (M3) and the others with better-prognosis disomy 3 (D3). However, we claim that not a single data alone but instead integrated omics data are powerful enough to explain the interplay of molecules and the biological phenotypes of cancer holistically (12–14). This motivates us to do this study in order to discover novel subgroups of UM patients that adopt an integrative approach.

**Figure 1:**
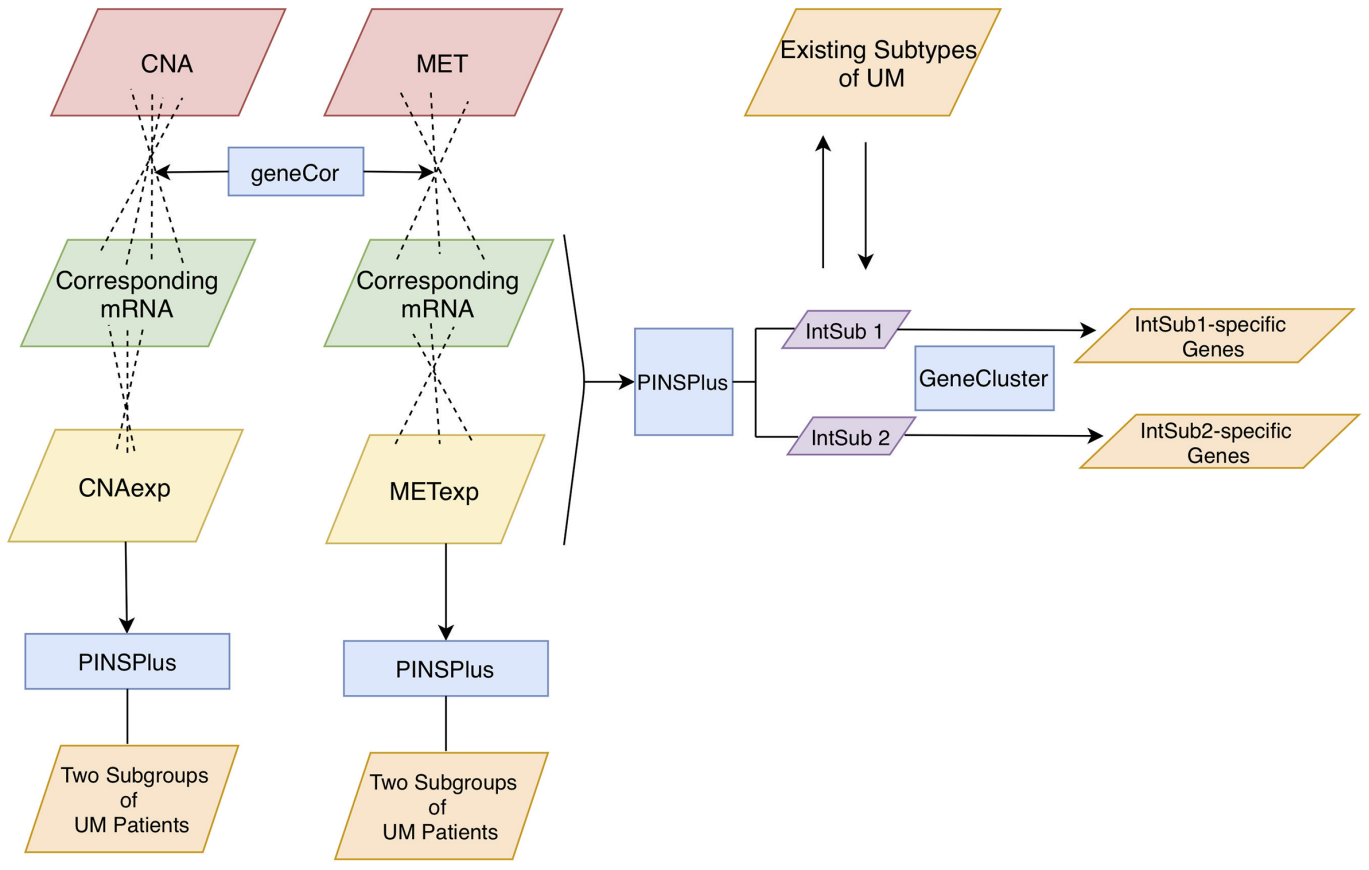
Analysis pipeline. Firstly, we inputted CNA and MET datasets with their corresponding mRNA data to the function geneCor to identify a list of CNAexp and METexp genes, respectively. Then, PINPlus was used to extract different patient subgroups for individual CNAexp and METexp datasets and integration of CNAexp + METexp + mRNA data through single and integrated analyses, respectively. Finally, we discovered subtype-specific genes within each identified integrated subgroup, IntSub1 and IntSub2, using the R package GeneCluster. UM, uveal melanoma.

In this study, we aimed to analyze three omics profiles, namely, CNA, MET, and mRNA, in a UM cohort from The Cancer Genome Atlas (TCGA). To this purpose, we identified the significant correlation between CNA and MET versus their own corresponding expression levels (**Figure 1**). It was of importance to note that the omics experiments were conducted with thousands of simultaneous hypothesis tests (15). Therefore, the adjusted *P*-value using the Benjamini–Hochberg procedure (16) as a measure of significant tests controlling the number of false discoveries was necessarily considered in this work. Then, single and joint analyses of the three data types were performed using the tool PINSPlus (17, 18). As a result, we discovered two novel integrative subgroups, IntSub1 and IntSub2, which could be potentially a future classification system for UM patients. These discoveries could influence the frequency of metastatic surveillance and support medical practitioners to choose an appropriate treatment regime.

## 2 Materials and Methods

### 2.1 Materials

The three datasets, namely, CNA, MET, and mRNA expression, were collected from the TCGA project (TCGA, Firehose Legacy) (5) and downloaded from the cBioPortal website (19, 20). The UM cohort is described in **Table 1**.

**Table 1:** Description of a cohort of UM patients used in the study.

Omics data	Platform	Description
mRNA	mRNA sequencing	A continuous matrix whose columns (the number of samples) are 80 samples and rows (the number of genes) are 20,440 genes
CNA	Affymetrix SNP6 Whole-exome sequencing	A discrete matrix whose columns (the number of samples) are 80 samples and rows (the number of genes) are 24,776 genes. There are four copy-number levels indicated for each gene, namely, −2, −1, 1, and 2. Two levels presented with minus value (i.e., −2, −1) show the loss level of copy-number compared with the two positive values (i.e., 1, 2) expressing the additional copies degree. For the 0 level, the gene is located in the diploid chromosomal region.
MET	Illumina Infinium HumanMethylation 450 platform	A continuous matrix whose columns (the number of samples) are 80 samples and rows (the number of genes) are 15,477 genes
Clinical data		Samples: 80 Overall survival (OS) status was defined as vital status (dead or alive), whereas OS time was identified as the time to UM death or last follow-up (unit: day). The follow-up time OS was truncated to 2,600 days.

### 2.2 Data Preprocessing

There were two preprocessing steps applied to the three profiles (i.e., mRNA, CNA, and MET) from the data. We first checked if the 80 patients from each of the three profiles and clinical data were matched. Then, we detected genes whose missing values were more than 50% using the *k*-nearest neighbor algorithm (21) from the CancerSubtypes package (version 1.14.0) (22).

### 2.3 Identification and Examination of the Relationship of CNAexp and METexp Genes

Here, we kept only genes shared between CNA and mRNA, as well as between MET and mRNA. To identify and examine the relationship of CNAexp and METexp genes, we used the R tool geneCor (14). Roughly, the tool first computed the correlation coefficients (*r*) between MET and mRNA, as well as between CNA and mRNA based on Spearman’s rank method, and then, the conversion of significant *r* (i.e., adjusted *P*-value ≤ 0.05; Benjamini–Hochberg (16); two-sided) into *Z* values by Fisher’s *Z*-transformation following the equation: *Z* = 0.5 ln[(1 + *r*)/(1 − *r*]). Secondly, the overall distributions of calculating *Z* values were pictured automatically. Thirdly, geneCor computed the skewness of the *Z*-score distributions using the D’Agostino test. The overall skewness illustrated whether CNA or MET was correlated positively or negatively with their own corresponding mRNA. Parallelly, geneCor also issued two sets of genes, CNAexp and METexp, whose CNA and MET significantly correlated with their corresponding mRNA expression levels, respectively. Further analysis was performed using FSbyCOX in the package CancerSubtypes (version 1.14.0) (22) to only retain a small number of genes associated significantly with a prognostic value (*P*-value ≤ 0.0005; log-rank test; two-tailed) in the two gene sets (i.e., CNAexp and METexp).

### 2.4 Single and Integrated Subtyping

The related study proposed by Robertson et al. (5) found the four different molecular groups based on highly expressed genes, CNA and MET, separately. We hypothesized that an integrative clustering analysis, comprising the three profiles above, would be a more powerful approach. Moreover, our clustering tool, PINSPlus (version 2.0.5) (17, 18), demonstrated its great ability in cancer subtyping, in general, using multi-omics data. Especially, it classified breast cancer patients into two subgroups that have possessed biologically and clinically meaningful properties (14). We, therefore, continued applying this tool to seeking the optimal group number of UV patients. In this study, we kept all the parameters of PINSPlus as default (i.e., clustering method was *k*-means); except for the number of candidate groups, *k* was set to a range from 2 to 10. The area under the receiver operating characteristic (AUC) value allowed us to choose the optimal *k*.

### 2.5 Subgroup-Specific Gene Determination and Enrichment Analysis

To observe the biological differences between identified UM subgroups, we sought to discover the subtype-specific genes using the package GeneCluster (version 0.1.0) (14). Given the lists of genes (i.e., METexp and CNAexp), this tool computed the mean expression level of each gene in each identified patient subgroup across all samples. Then, the gene whose mean expression value was the highest will be allotted to a cluster if the *P*-value ≤0.05 (one-way ANOVA test; two-sided). Finally, the gene will be recognized officially as belonging to that subtype if the adjusted *P*-value ≤0.05 (Benjamini–Hochberg procedure (16); two-tailed).

Subsequently, in order to investigate further the biological themes from the gained subgroup-specific genes, we implemented the enrichment analysis using the DAVID tool (version 6.8) (23, 24). Also, the output was concentrated into functional-related gene groups or different meaningful terms that were convenient to translate into the clinic. The significance levels of these terms were assessed based on *P*-value (Fisher’s exact test). In other words, a list of genes with a smaller *P*-value was more overrepresented and had a stronger association to the subtype phenotypes.

## 3 Results

### 3.1 Identification and Examination of the Relationship of CNAexp and METexp Genes

**Figure 2:**
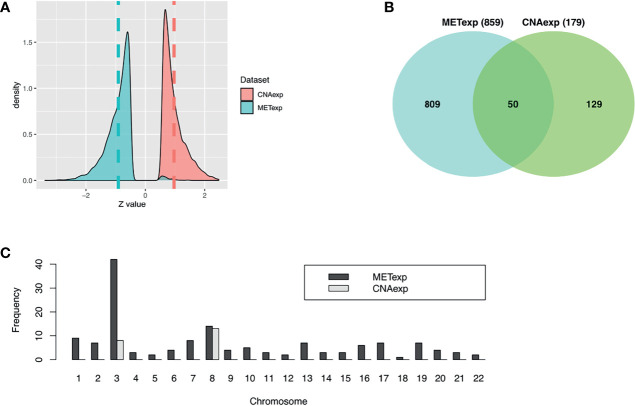
Characteristics of CNAexp and METexp in UM. **(A)** Two *Z*-score distributions showed two associations of MET or CNA with their respective mRNA. **(B)** Intersection between 859 METexp genes and 179 CNAexp genes. **(C)** Side-by-side bar chart showed the frequency of the CNAexp or METexp genes against the total count of genes in each chromosome arm. CNA, DNA copy number aberrations; MET, epigenetic DNA methylation.

Our tool geneCor provided us with the two sets comprising 4,139 CNAexp genes and 8,157 METexp genes (see **Supplementary Table S1**). As pictured in **Figure 2A**, the CNAexp genes were significantly skewed to the right (skewness = 1.3511, *P*-value < 2.2 × 10^−16^; D’Agostino test; two-sided) consistent with the results reported in (25), while the METexp genes were significantly skewed to the left (skewness = −0.3419, *P*-value < 2.2 × 10^−16^; D’Agostino test; two-sided) consistent with the results reported in (26). This indicated that there was a consistently converse relation of mRNA with CNA and MET genes. As mentioned, we truncated genes per the gene set above (i.e., CNAexp and METexp) based on the association with the OS of patients. Particularly, due to an overwhelming number of genes in each set, we only preserved genes per set if *P*-value <0.0005. Finally, 179 CNAexp genes and 859 METexp genes were obtained. It was a weak intersection (50 genes) between CNAexp and METexp, indicating that the CNAexp and METexp were two poorly non-disjoint events (**Figure 2B**). **Figure 2C** shows the frequency of the CNAexp or METexp genes against the total count of genes in each chromosome arm. Of particular interest, CNAexp only distributed in two chromosomes 3 and 8, especially almost in chromosome 8, implying not only a poor prognosis but also a considerably reduced survival (27–30). Also, we could observe that the METexp genes displayed a regional genomic preference for MET, particularly on chromosome 3, involving in high metastatic risk (26).

### 3.2 Single and Integrated Subtyping

**Figure 3:**
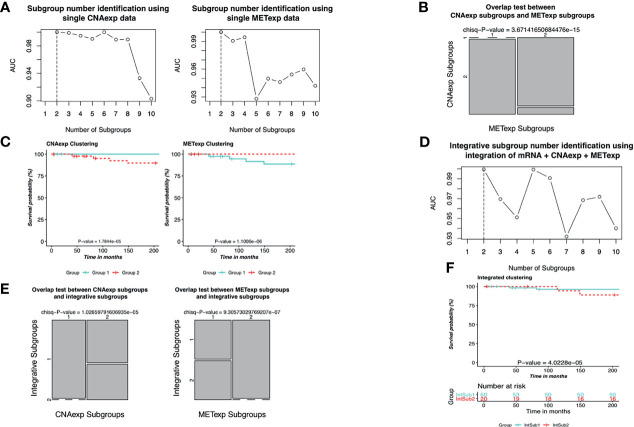
Identification of UM molecular subgroups using individual CNAexp and METexp genes for single clustering and mRNA + CNAexp + METexp for joint clustering. **(A, D)** AUC values obtained for each value of *k*. The optimal *k* has the highest AUC value, in which (**A**—left, **A**–right, and **D**) the results are of CNAexp alone, METexp alone, and integration of mRNA + CNAexp + METexp, respectively. **(B)** Overlap test between subgroups of CNAexp and METexp. **(E)** Overlap test between integrative subgroups versus CNAexp subgroups (left) and versus METexp subgroups (right). **(C, F)** Kaplan–Meier survival curves for the CNAexp subgroups (**C**—left), METexp subgroups (**C**—right), and **(F)** integrated subgroups.

As described in the *Materials and Methods* section, we implemented the single clustering analyses for CNAexp and METexp, separately. For METexp, the *k* of two with the AUC of 1.0000 was optimal (**Figure 3A**). Similarly, for CNAexp, the same *k* and AUC were also optimal again (**Figure 3A**). Notably, the number of patients assigned to either of the two CNAexp subgroups significantly overlapped with that of the two METexp subgroups (*P*-value = 3.6714 × 10^−15^; *χ* ^2^ test; two-sided; **Figure 3B**). The heatmap shows the expression patterns of CNAexp subgroups and METexp subgroups from integrated analysis by PINSPlus (**Supplementary Figure S1**). Moreover, the association between our integrated subgroups, IntSub1 and IntSub2, versus patient subtypes in (5) using mRNA data is also shown in **Supplementary Figure S2**. Interestingly, IntSub1 was divided almost into subgroups 1 to 3, whereas most patients in IntSub2 belonged previously to subtype 4. We then employed the survival analysis for the acquired subgroups of CNAexp and METexp. The two CNAexp subgroups were revealed to be statistically meaningful to the OS (*P*-value = 1.7844 × 10^−5^; two-sided; **Figure 3C**). Also, with the Cox *P*-value = 1.1006 × 10^−6^, the two METexp subgroups were significantly correlated with the OS (**Figure 3C**). These results told us that the data single clustering strategy seemed to be effective in this case. However, the given single analyses might only show the results that reflected the solitary aberration in UM pathology.

Next, the integrative clustering analysis was leveraged for a combination of CNAexp, METexp, and mRNA gene sets in a similar manner with the single clustering analysis above. Interestingly, PINSPlus classified UM patients into two integrative subgroups called IntSub1 (*n* = 60) and IntSub2 (*n* = 20) (**Figure 3D**). Specially, they were consistent significantly with the single subgroups of the CNAexp dataset (*P*-value = 1.0266 × 10^−5^; *χ* ^2^ test; two-sided; **Figure 3E**) and the METexp dataset (*P*-value = 9.3057 × 10^−7^; *χ* ^2^ test; **Figure 3E**). On top of that, we then investigated the survival analysis which revealed that the two integrated subgroups possessed statistically different factors for the survival of UM patients (*P*-value = 4.0228 × 10^−5^; log-rank test; **Figure 3F**). Also, in **Figure 3F**, the patients in IntSub2 were significantly worse than those in IntSub1 (hazard ratio of 6.1204 and 95% confidence interval between 2.5970 and 14.4200; IntSub1 was reference; log-rank test). Also, we reviewed the statistical descriptions for UM patients, containing age, gender, tumor stages, and metastasis status, between the IntSub1 and IntSub2 provided in **Supplementary Table S2**. These results bolstered our confidence in the effectiveness of our previous strategy (14) in discovering the novel UM patient subgroups under the perspective of integration.

### 3.3 Molecular Characteristics of Integrated Subgroups

#### 3.3.1 Determination of Subgroup-Specific Genes

As mentioned earlier, the GeneCluster tool was leveraged to exploit subtype-specific gene lists. Accordingly, we extracted three subgroup-specific gene lists for the two integrative subgroups using three kinds of profiles: mRNA, CNAexp, and METexp. Specifically, these lists were established on average mRNA expression levels (IntSub1: 347 genes and IntSub2: 431 genes; **Supplementary Table S3**), average CNA aberrations (IntSub1: 108 genes and IntSub2: 71 genes; **Supplementary Table S4**), and average MET aberrations (IntSub1: 492 and IntSub2: 345 genes; **Supplementary Table S5**). Notably, we checked the intersection of the subgroup-specific genes from mRNA with UM immune single-cell gene signature from Durante et al. (31) and revealed that 46 overlapped genes (13.26%) in IntSub1 belonged to B-cell cluster, CD4 T follicular helper cluster, M2 macrophage cluster, Mitotic CD8 T-cell cluster, etc. (**Supplementary Table S6**). Meanwhile, 107 overlapped genes (24.82%) in IntSub2 were associated with immune cells such as B cells, CD4 T follicular helper, CD8, gamma delta T cells, and mitotic CD8 T cells (**Supplementary Table S6**). This indicated that the UM pathology had a strong connection to the abnormally expressed genes related to immune cells. Interestingly, we found that that the highest expressed gene based on copy number aberrations, *SLCO5A1*, was identified to associate with poor outcome (32), which could be a prospective interpretation for the worse prognosis of IntSub2 patients compared with those in IntSub1. Notably, *SLCO5A1* was considered as a prognosis gene correlated with the immune infiltrates. The immune cell infiltration level was noted to be a crucial factor in predicting the UM prognosis (33). Supplementally, we sought out that *BAP1* was associated with abnormal DNA methylation within IntSub2 samples rather than other subtypes. It was reported that about 22% of familial UM cases found the muted *BAP1. BAP1* mutations raised not only a large tumor diameter percentage but also the metastasis risk in UM patients. This indicated that *BAP1* testing is a reasonable recommendation for hereditary melanoma (34). Additionally, *PTP4A3*, the most overexpressed gene ranked by mean expression value among specific genes of IntSub2, was defined as a marker of poor prognosis involved in cell migration and metastatic progression (35). Furthermore, metastasis is a confident signal of the poor outcome, resulting in death in most UM cases (36).

#### 3.3.2 Enrichment Analysis Using the DAVID Tool

We next performed the enrichment analysis as described above with the given subgroup-specific genes. Remarkably, the top biological processes for IntSub1-specific CNAexp genes included endonuclease activity and interleukin-17 receptor activity and transcription factor binding (**Supplementary Table S7** and **Supplementary Figure S3**); IntSub1-specific METexp genes were associated with the positive regulation of cell migration, immune effector process, and positive regulation of hydrolase activity (**Supplementary Table S8** and **Supplementary Figure S4**). Conversely, the IntSub2 was characterized most in cellular cation homeostasis embracing, especially, the regulation of pH and the regulation of calcium ion in the CNAexp profile (**Supplementary Table S7** and **Supplementary Figure S5**). Also, the IntSub2 was distinguished by common abnormalities of METexp genes related to the regulation of gene expression and cellular macromolecule biosynthetic process (**Supplementary Table S8** and **Supplementary Figure S6**).

In this study, we also compared the subgroup-specific genes from the two lists: mRNA (**Supplementary Table S3**) and CNAexp (**Supplementary Table S4**) with the FoundationOne CDx (updated on June 15, 2020) that included 321 genes relating closely to cancer and participating in the process of tumorigenesis. Consequently, we revealed 22 subgroup-specific mRNA expression genes (bold red gene names in **Supplementary Table S3**) and eight subgroup-specific CNAexp genes (bold red gene names in **Supplementary Table S4**) included in the database above. Collectively, our results reinforced the clinical association between the obtained subgroup-specific genes and melanoma formation.

#### 3.3.3. Prognostic Factor Identification

**Figure 4:**
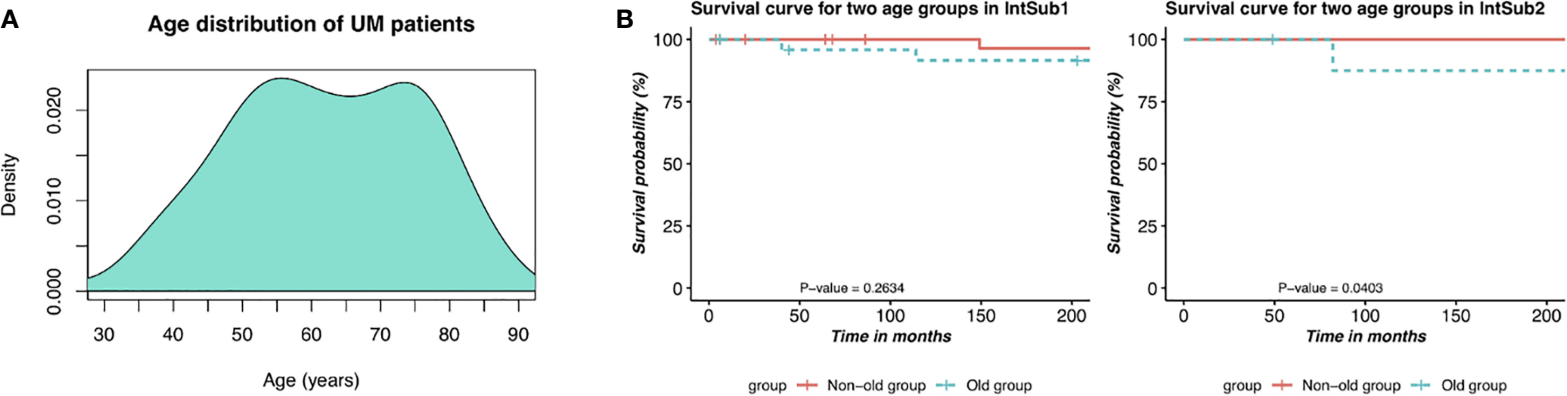
Description of prognostic risks of UM patients in each integrative subtype. **(A)** Bimodal age distribution of UM patients. **(B)** Kaplan–Meier survival curves of patients in the two age groups in the two identified subgroups, IntSub1 and IntSub2.

We then sought to conduct the age at diagnosis and survival time analyses in order to define the prognosis factor of two UM subtypes. The results are shown in **Table 2**. It is worth mentioning that 60-year-old or older patients were highly risky to have UM. In addition, there was a distinct difference in the average survival day between IntSub1 and IntSub2 patients: 885.2667 and 617.0000 days, respectively. This indicated that the OS of UM patients could be foreknown dependent partly on which subgroup a patient is assigned to, to some extent. Besides, the patients in the IntSub1 were characterized by the average age of 60.3333 as well as the average OS of 885.2667 days, whereas those numbers in the IntSub2 were 65.6000 years old and 617.0000 days. Obviously, although the average age of the patients in the IntSub1 was only 5 years younger than that of their counterparts in IntSub2, they could live about 9 months longer than the patients in IntSub2. These results should be understood that age-related risks and survival rates might be separate in these integrative subgroups. For a better understanding, we took into account the risk of the two age groups in each subgroup comprising the mid-adults (21–65 years) and the older adults (>65 years) from the 80 UM patients (22–86 years old) in the clinical data. The reason we chose the threshold of 65 years old was because **Figure 4A** illustrates a bimodal age distribution, implying that we had two groups naturally.

**Table 2:** Average diagnosis ages and survival time of the UM patients in the two integrated subgroups.

	IntSub1	IntSub2
Average age (years)	60.3333	65.6000
Average survival time (days)	885.2667	617.0000

The two age groups, the non-old group and the old group, in each subgroup were interrogated by the survival analyses. Observing the results reported in **Figure 4B**, we revealed a significant survival difference between the two age groups in the IntSub2, whereas no statistical significance in patient outcome between the two age groups was seen in the IntSub1, indicating that age factor could be a risk factor to predict the survival time.

## 4 Discussion and Conclusion

Recently, genomic profiling at multiple levels (e.g., genomics, epigenomics, transcriptomics) has been boomed (37). The abundant omics type of data has been easily accessed from public databases like TCGA facilitating a better understanding of molecular events behind cancer progression. Additionally, based on the associations between the three types of omics data (mRNA, MET, and CNA), we successfully classified breast cancer into two patient subsets which improved the weak manifestations of the intrinsic subtypes, especially in association with the biological phenotype in a prior work. With these concerns in mind, we have decided to apply this successful framework to a rare cancer like UM.

Here, we defined the two lists of CNAexp and METexp based on the correlations of CNA and MET with their mRNA at first. The resulting lists are leveraged to stratify not only individually but also integratively the 80 UM patients using the PINSPlus tool. We revealed the two molecular subgroups (IntSub1 and IntSub2) along with their subtype-specific genes that help to uncover significantly different clinical characteristics as well as patient outcomes. Importantly, there existed several poorly prognostic genes (*SLCO5A1*, *BAP1*, and *PTP4A3*) which could lead to shorter OS of IntSub2 patients. We next recruited the DAVID tool to perform the enrichment analysis in each integrated clustering. Notably, the IntSub1 showed the overexpression of genes enriched significantly in the immune system process (**Supplementary Table S4**). Besides, the IntSub2 displayed CNAexp genes known to be key factors in cellular cation homeostasis and regulation of calcium. These findings are likely to help oncologists and physicists find out distinct treatment strategies for the two subgroups.

The finding of these subgroups could be a suggestion in clinical application for UM treatment. For example, in the IntSub1, the IL-17 (*IL17RE*, *IL17RD*, and *IL17RC*) played vital roles in immune responses which stimulated the tumor growth and repressed the antitumor activity (38). Fabre et al. (39) affirmed in their study that the IL-17/IL-17R axis could be a novel immunotherapeutic target relevant to the antitumor purpose. Besides, the dense appearance of mutated genes is enriched in the cellular cation homeostasis group (i.e., K^+^, Ca^2+^, Na^+^, and H^+^). Cell proliferation and apoptosis were regulated by various cation channels. For instance, K^+^ channels participated in the stimulation of the cell end, thus declining the cell number. Therefore, the changeable potassium channels contributed to the malignant expression of cancer (40). In the cellular cation homeostasis gene group, *SGK3* played an activation role of potassium channels (41). Moreover, several prior studies showed the promising therapy of K^+^ channel blocking in cancer treatment. This enhanced the consideration of using drugs inhibiting the potassium channels as chemotherapy for UM patients. As an example, astemizole was repositioned in its use by blocking the *EAG1* channel which was one of the major potassium channels and brought remarkable efficacy for cancer cell growth (42). Alternatively, the small molecule which was able to block, inhibit, or regulate the calcium ion transport was reported to be a potential anticancer drug, such as brilliant blue G, oxidized ATP for melanoma cases (43). Taken together, targeted therapies may be efficient for the IntSub1 subgroup, while the combination of the cation channel blocker and chemotherapeutic drugs has the potential for IntSub2 patients.

In addition, we saw that the baselines of both IntSub1 and IntSub2 subgroups varied depending potentially on several clinical features being vital factors for prognosis. Thus, the survival comparison between the two subgroups was further interrogated by utilizing a multivariate Cox regression model in terms of age groups, tumor stages, gender, and histology cell type comparisons. The analysis results are shown in **Supplementary Table S8**. As a consequence, old age groups, tumor stage IV, and histology cell type comparison between spindle cell and predominant mixed spindle cell were considered as significantly independent prognostic factors.

Furthermore, some powerful predictive genes (except *BAP1*) for prognosis used in clinical routine in UM are not identified by our strategy. This can be regarded as a potential restriction of our work when deliberately leveraging the power of integration of multi-omics data. The following are several factors giving rise to the poor performance of our strategy. The first factor can be the “curse of dimensionality” being a typical problem when using multimodal data. Another factor can be possibly due to the different nature of data types. Most of the statistical tools only work well on continuous data, whereas the minority of them do well on discrete data. In this study, we have combined the two types.

In conclusion, multi-omics data integration contributes to dealing with the bottleneck in getting insights into complex multi-mechanism diseases like cancer in general and UM in particular. We determined the two clinically and molecularly distinct integrative subgroups, IntSub1 and IntSub2, which not only can be a potential alternative classification system in the future but also give more effective suggestions for UM treatment.

## Data Availability Statement

The original contributions presented in the study are included in the article/**Supplementary Material**. Further inquiries can be directed to the corresponding authors.

## Author Contributions

THYN drafted the manuscript, which was edited by all co-authors. Q-HN conceived and designed the approach, coded the geneCor and GeneCluster tools, and wrote the R codes for analyses. THYN ran the codes. THYN and Q-HN analyzed output data. TN coded the PINSPlus algorithm. Q-HN and D-HL jointly directed and supervised the work. All authors contributed to the article and approved the submitted version.

## Funding

This work was partially supported by NIH NIGMS under grant number GM103440 and by NSF under grant numbers 2001385 and 2019609. This research is also supported by Vingroup Innovation Foundation (VINIF) in project code VINIF.2019.DA18.

## Conflict of Interest

Authors THYN, Q-HN and D-HL were employed by Vingroup Big Data Institute.

The remaining authors declare that the research was conducted in the absence of any commercial or financial relationships that could be construed as a potential conflict of interest.

## Publisher’s Note

All claims expressed in this article are solely those of the authors and do not necessarily represent those of their affiliated organizations, or those of the publisher, the editors and the reviewers. Any product that may be evaluated in this article, or claim that may be made by its manufacturer, is not guaranteed or endorsed by the publisher.

## References

[1] Shields CL Furuta M Thangappan A Nagori S Mashayekhi A Lally DR. Metastasis of Uveal Melanoma Millimeter-by-Millimeter in 8033 Consecutive Eyes. Arch Ophthalmol (2009) 127(8):989–98. doi: 10.1001/archophthalmol.2009.208 19667335

[2] Anbunathan H Verstraten R Singh AD Harbour JW Bowcock AM. Integrative Copy Number Analysis of Uveal Melanoma Reveals Novel Candidate Genes Involved in Tumorigenesis Including a Tumor Suppressor Role for PHF10/BAF45a. Clin Cancer Res (2019) 25(16):5156. doi: 10.1158/1078-0432.CCR-18-3052 31227497 PMC6697622

[3] Kaliki S Shields CL. Uveal Melanoma: Relatively Rare But Deadly Cancer. Eye (London England) (2017) 31(2):241–57. doi: 10.1038/eye.2016.275 PMC5306463 27911450

[4] Yang Z-K Yang J-Y Xu Z-Z Yu W-H. DNA Methylation and Uveal Melanoma. Chin Med J (2018) 131(7):845–51. doi: 10.4103/0366-6999.228229 PMC5887744 29578129

[5] Robertson AG Shih J Yau C Gibb EA Oba J Mungall KL. Integrative Analysis Identifies Four Molecular and Clinical Subsets in Uveal Melanoma. Cancer Cell (2017) 32(2):204–20.e15. doi: 10.1016/j.ccell.2017.07.003 28810145 PMC5619925

[6] Barbagallo C Caltabiano R Broggi G Russo A Puzzo L Avitabile T. LncRNA LINC00518 Acts as an Oncogene in Uveal Melanoma by Regulating an RNA-Based Network. Cancers (Basel) (2020) 12(12):3867. doi: 10.3390/cancers12123867 PMC7767460 33371395

[7] Damato B Dopierala J Klaasen A van Dijk M Sibbring J Coupland SE. Multiplex Ligation-Dependent Probe Amplification of Uveal Melanoma: Correlation With Metastatic Death. Invest Ophthalmol Visual Sci (2009) 50(7):3048–55. doi: 10.1167/iovs.08-3165 19182252

[8] Kilic E van Gils W Lodder E Beverloo HB van Til ME Mooy CM. Clinical and Cytogenetic Analyses in Uveal Melanoma. Invest Ophthalmol Visual Sci (2006) 47(9):3703–7. doi: 10.1167/iovs.06-0101 16936076

[9] Vivet-Noguer R Tarin M Roman-Roman S Alsafadi S. Emerging Therapeutic Opportunities Based on Current Knowledge of Uveal Melanoma Biology. Cancers (Basel) (2019) 11(7):1019. doi: 10.3390/cancers11071019 PMC6678734 31330784

[10] Russo D Di Crescenzo RM Broggi G Merolla F Martino F Varricchio S. Expression of P16INK4a in Uveal Melanoma: New Perspectives. Front Oncol (2020) 10:562074. doi: 10.3389/fonc.2020.562074 33154942 PMC7590828

[11] Broggi G Ieni A Russo D Varricchio S Puzzo L Russo A. The Macro-Autophagy-Related Protein Beclin-1 Immunohistochemical Expression Correlates With Tumor Cell Type and Clinical Behavior of Uveal Melanoma. Front Oncol (2020) 10:589849. doi: 10.3389/fonc.2020.589849 33330070 PMC7714947

[12] Subramanian I Verma S Kumar S Jere A Anamika K. Multi-Omics Data Integration, Interpretation, and Its Application. Bioinf Biol Insights (2020) 14:1177932219899051–. doi: 10.1177/1177932219899051 PMC7003173 32076369

[13] Shen R Mo Q Schultz N Seshan VE Olshen AB Huse J. Integrative Subtype Discovery in Glioblastoma Using Icluster. PloS One (2012) 7(4):e35236. doi: 10.1371/journal.pone.0035236 22539962 PMC3335101

[14] Nguyen Q-H Nguyen H Nguyen T Le D-H. Multi-Omics Analysis Detects Novel Prognostic Subgroups of Breast Cancer. Front Genet (2020) 11:574661. doi: 10.3389/fgene.2020.574661 33193681 PMC7594512

[15] Dunkler D Sanchez-Cabo F Heinze G. Statistical Analysis Principles for Omics Data. Methods Mol Biol (Clifton NJ) (2011) 719:113–31. doi: 10.1007/978-1-61779-027-0_5 21370081

[16] Benjamini Y Hochberg Y. Controlling the False Discovery Rate: A Practical and Powerful Approach to Multiple Testing. J R Stat Soc Ser B (Methodological) (1995) 57(1):289–300. doi: 10.1111/j.2517-6161.1995.tb02031.x

[17] Nguyen H Shrestha S Draghici S Nguyen T. PINSPlus: A Tool for Tumor Subtype Discovery in Integrated Genomic Data. Bioinf (Oxford England) (2018) 35(16):2843–6. doi: 10.1093/bioinformatics/bty1049 30590381

[18] Nguyen T Tagett R Diaz D Draghici S. A Novel Approach for Data Integration and Disease Subtyping. Genome Res (2017) 27(12):2025–39. doi: 10.1101/gr.215129.116 PMC5741060 29066617

[19] Cerami E Gao J Dogrusoz U Gross BE Sumer SO Aksoy BA. The Cbio Cancer Genomics Portal: An Open Platform for Exploring Multidimensional Cancer Genomics Data. Cancer Discov (2012) 2(5):401–4. doi: 10.1158/2159-8290.CD-12-0095 PMC3956037 22588877

[20] Gao J Aksoy BA Dogrusoz U Dresdner G Gross B Sumer SO. Integrative Analysis of Complex Cancer Genomics and Clinical Profiles Using the Cbioportal. Sci Signal (2013) 6(269):pl1. doi: 10.1126/scisignal.2004088 23550210 PMC4160307

[21] Batista GEAPA Monard MC. A Study of K-Nearest Neighbour as an Imputation Method. HIS (2002) 87:251–60.

[22] Xu T Le TD Liu L Su N Wang R Sun B. CancerSubtypes: An R/Bioconductor Package for Molecular Cancer Subtype Identification, Validation and Visualization. Bioinf (Oxford England) (2017) 33(19):3131–3. doi: 10.1093/bioinformatics/btx378 28605519

[23] Huang da W Sherman BT Lempicki RA. Systematic and Integrative Analysis of Large Gene Lists Using DAVID Bioinformatics Resources. Nat Protoc (2009) 4(1):44–57. doi: 10.1038/nprot.2008.211 19131956

[24] Huang da W Sherman BT Lempicki RA. Bioinformatics Enrichment Tools: Paths Toward the Comprehensive Functional Analysis of Large Gene Lists. Nucleic Acids Res (2009) 37(1):1–13. doi: 10.1093/nar/gkn923 19033363 PMC2615629

[25] Shao X Lv N Liao J Long J Xue R Ai N. Copy Number Variation is Highly Correlated With Differential Gene Expression: A Pan-Cancer Study. BMC Med Genet (2019) 20(1):175. doi: 10.1186/s12881-019-0909-5 31706287 PMC6842483

[26] Field MG Kuznetsov JN Bussies PL Cai LZ Alawa KA Decatur CL. BAP1 Loss Is Associated With DNA Methylomic Repatterning in Highly Aggressive Class 2 Uveal Melanomas. Clin Cancer Res (2019) 25(18):5663. doi: 10.1158/1078-0432.CCR-19-0366 31285370 PMC6744995

[27] Hammond DW Al-Shammari NS Danson S Jacques R Rennie IG Sisley K. High-Resolution Array CGH Analysis Identifies Regional Deletions and Amplifications of Chromosome 8 in Uveal Melanoma. Invest Ophthalmol Vis Sci (2015) 56(6):3460–6. doi: 10.1167/iovs.14-16215 26030101

[28] Sisley K Rennie IG Parsons MA Jacques R Hammond DW Bell SM. Abnormalities of Chromosomes 3 and 8 in Posterior Uveal Melanoma Correlate With Prognosis. Genes Chromosom Cancer (1997) 19(1):22–8. doi: 10.1002/(SICI)1098-2264(199705)19:1<22::AID-GCC4>3.0.CO;2-2 9135991

[29] Versluis M de Lange MJ van Pelt SI Ruivenkamp CA Kroes WG Cao J. Digital PCR Validates 8q Dosage as Prognostic Tool in Uveal Melanoma. PloS One (2015) 10(3):e0116371. doi: 10.1371/journal.pone.0116371 25764247 PMC4357379

[30] van den Bosch T van Beek JG Vaarwater J Verdijk RM Naus NC Paridaens D. Higher Percentage of FISH-Determined Monosomy 3 and 8q Amplification in Uveal Melanoma Cells Relate to Poor Patient Prognosis. Invest Ophthalmol Vis Sci (2012) 53(6):2668–74. doi: 10.1167/iovs.11-8697 22427574

[31] Durante MA Rodriguez DA Kurtenbach S Kuznetsov JN Sanchez MI Decatur CL. Single-Cell Analysis Reveals New Evolutionary Complexity in Uveal Melanoma. Nat Commun (2020) 11(1):496. doi: 10.1038/s41467-019-14256-1 31980621 PMC6981133

[32] Luo H Ma C. Identification of Prognostic Genes in Uveal Melanoma Microenvironment. PloS One (2020) 15(11):e0242263–e. doi: 10.1371/journal.pone.0242263 PMC7668584 33196683

[33] Narasimhaiah D Legrand C Damotte D Remark R Munda M De Potter P. DNA Alteration-Based Classification of Uveal Melanoma Gives Better Prognostic Stratification Than Immune Infiltration, Which has a Neutral Effect in High-Risk Group. Cancer Med (2019) 8(6):3036–46. doi: 10.1002/cam4.2122 PMC6558590 31025552

[34] Rai K Pilarski R Boru G Rehman M Saqr AH Massengill JB. Germline BAP1 Alterations in Familial Uveal Melanoma. Genes Chromosom Cancer (2017) 56(2):168–74. doi: 10.1002/gcc.22424 PMC5490375 27718540

[35] Duciel L Anezo O Mandal K Laurent C Planque N Coquelle FM. Protein Tyrosine Phosphatase 4A3 (PTP4A3/PRL-3) Promotes the Aggressiveness of Human Uveal Melanoma Through Dephosphorylation of CRMP2. Sci Rep (2019) 9(1):2990. doi: 10.1038/s41598-019-39643-y 30816227 PMC6395723

[36] Lane AM Kim IK Gragoudas ES. Survival Rates in Patients After Treatment for Metastasis From Uveal Melanoma. JAMA Ophthalmol (2018) 136(9):981–6. doi: 10.1001/jamaophthalmol.2018.2466 PMC6142974 29955797

[37] Chakraborty S Hosen MI Ahmed M Shekhar HU. Onco-Multi-OMICS Approach: A New Frontier in Cancer Research. BioMed Res Int (2018) 2018:9836256. doi: 10.1155/2018/9836256 30402498 PMC6192166

[38] Yang B Kang H Fung A Zhao H Wang T Ma D. The Role of Interleukin 17 in Tumour Proliferation, Angiogenesis, and Metastasis. Mediators Inflamm (2014) 2014:623759–. doi: 10.1155/2014/623759 PMC4119694 25110397

[39] Fabre J Giustiniani J Garbar C Antonicelli F Merrouche Y Bensussan A. Targeting the Tumor Microenvironment: The Protumor Effects of IL-17 Related to Cancer Type. Int J Mol Sci (2016) 17(9):1433. doi: 10.3390/ijms17091433 PMC5037712 27589729

[40] Comes N Serrano-Albarrás A Capera J Serrano-Novillo C Condom E Ramón YCS. Involvement of Potassium Channels in the Progression of Cancer to a More Malignant Phenotype. Biochim Biophys Acta (2015) 1848(10 Pt B):2477–92. doi: 10.1016/j.bbamem.2014.12.008 25517985

[41] Gamper N Feng Y Friedrich B Lang PA Henke G Huber SM. K+ Channel Activation by All Three Isoforms of Serum- and Glucocorticoid-Dependent Protein Kinase SGK. Pflugers Arch (2002) 445(1):60–6. doi: 10.1007/s00424-002-0873-2 12397388

[42] Downie BR Sánchez A Knötgen H Contreras-Jurado C Gymnopoulos M Weber C. Eag1 Expression Interferes With Hypoxia Homeostasis and Induces Angiogenesis in Tumors. J Biol Chem (2008) 283(52):36234–40. doi: 10.1074/jbc.M801830200 PMC2606018 18927085

[43] Babcock JJ Du F Xu K Wheelan SJ Li M. Integrated Analysis of Drug-Induced Gene Expression Profiles Predicts Novel hERG Inhibitors. PloS One (2013) 8(7):e69513–e. doi: 10.1371/journal.pone.0069513 PMC3720659 23936032

