# Supplementary material for: Re-Identification of Patient Subgroups in Uveal Melanoma
Source: Front Oncol. 2021 Oct 20;11:731548. doi: 10.3389/fonc.2021.731548 (PMC8564479; doi:10.3389/fonc.2021.731548)
Supplement: Supplementary file 1 [file Image_1.pdf]

Supplementary Figure

Re-identification of patient subgroups in Uveal Melanoma

Yen T.H. Nguyen[1,†], Tin Nguyen[2], Quang-Huy Nguyen[1,†*], Duc-Hau Le[1,3*]

[1]Department of Computational Biomedicine, Vingroup Big Data Institute, Hanoi, Vietnam.

[2]Department of Computer Science and Engineering, University of Nevada, Reno, NV, USA.

[3]College of Engineering and Computer Science, VinUniversity, Hanoi, Vietnam.

† these authors have contributed equally

* To whom correspondence should be addressed. Tel: (84)912324564; Email: huynguyen96.dnu@gmail.com, hauldhut@gmail.com.

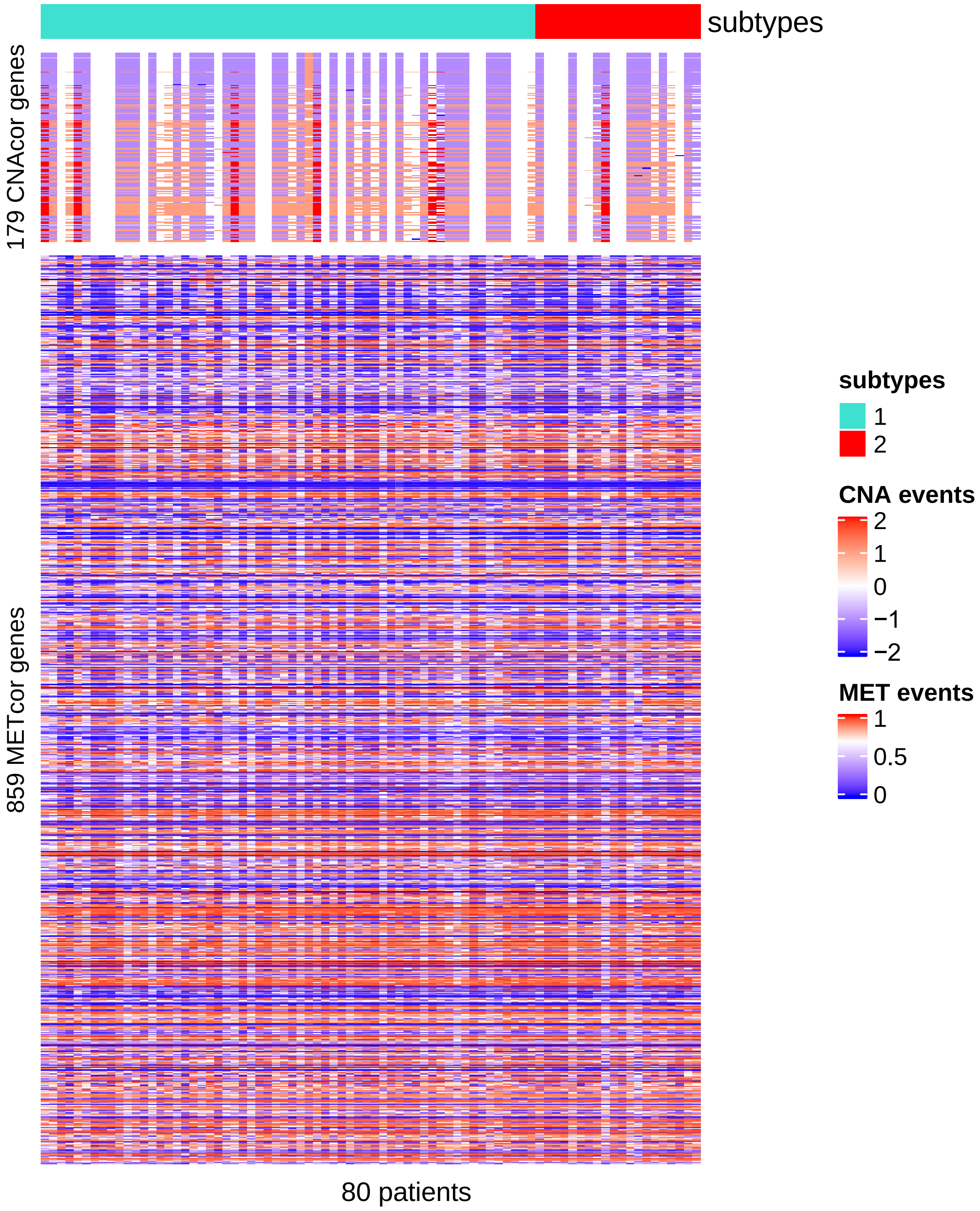

Supplementary Figure 1. Heatmaps that show the expression patterns of CNAexp subgroups (mid) and METexp subgroups (bottom) from integrated analysis by PINSPlus.

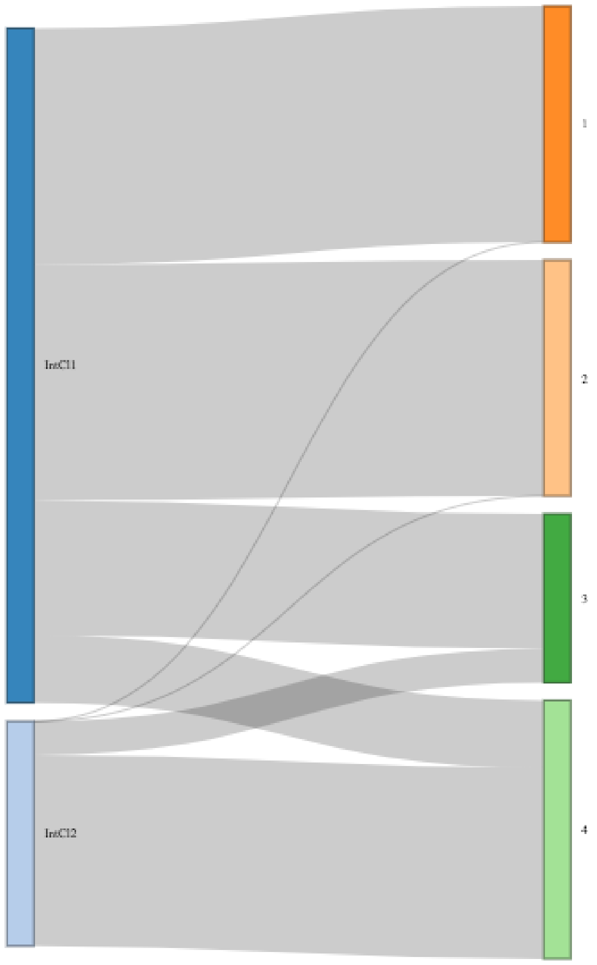

Supplementary Figure 2. Association between IntSub1 and IntSub2 subgroups (left) versus TCGA UVM classification [1] (right) using mRNA.

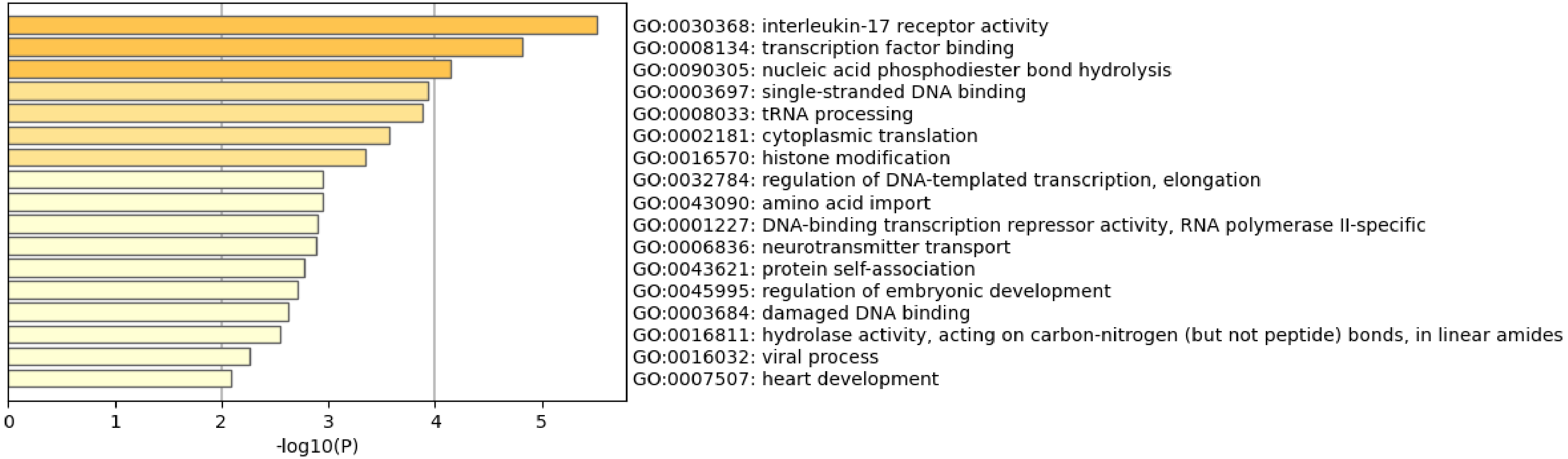

Supplementary Figure 3. Enrichment analysis with subtype-specific genes from CNAexp in IntSub1.

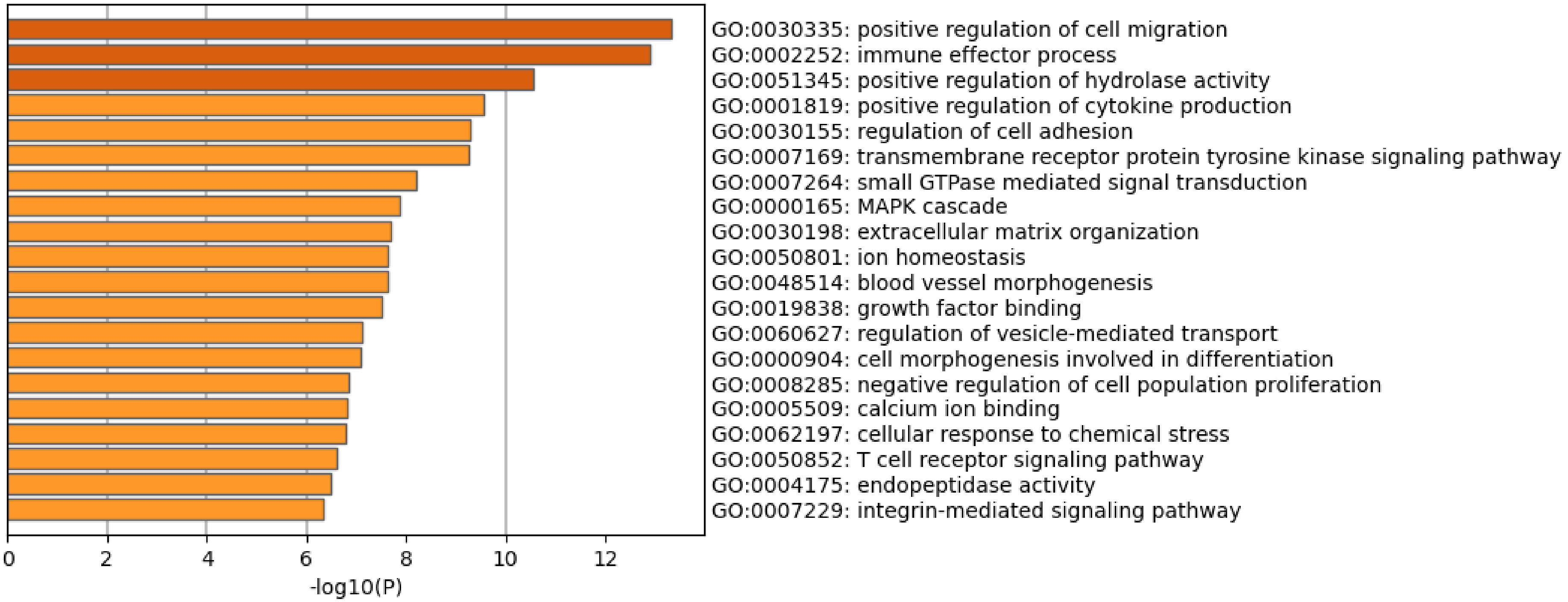

Supplementary Figure 4. Enrichment analysis with subtype-specific genes from METexp in IntSub1.

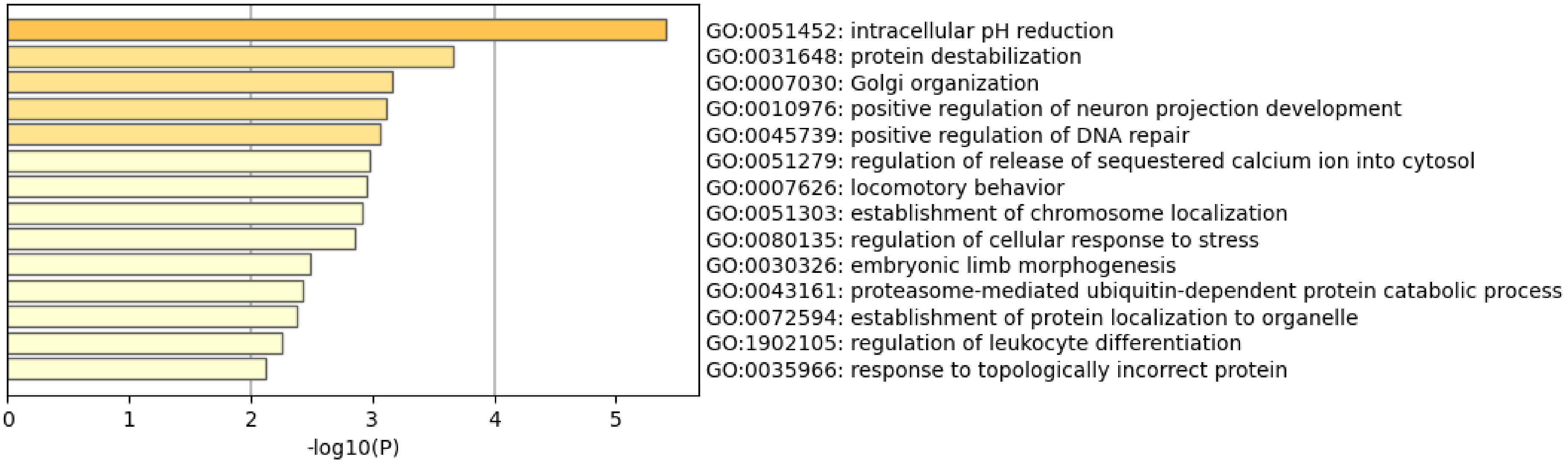

Supplementary Figure 5. Enrichment analysis with subtype-specific genes from CNAexp in IntSub2.

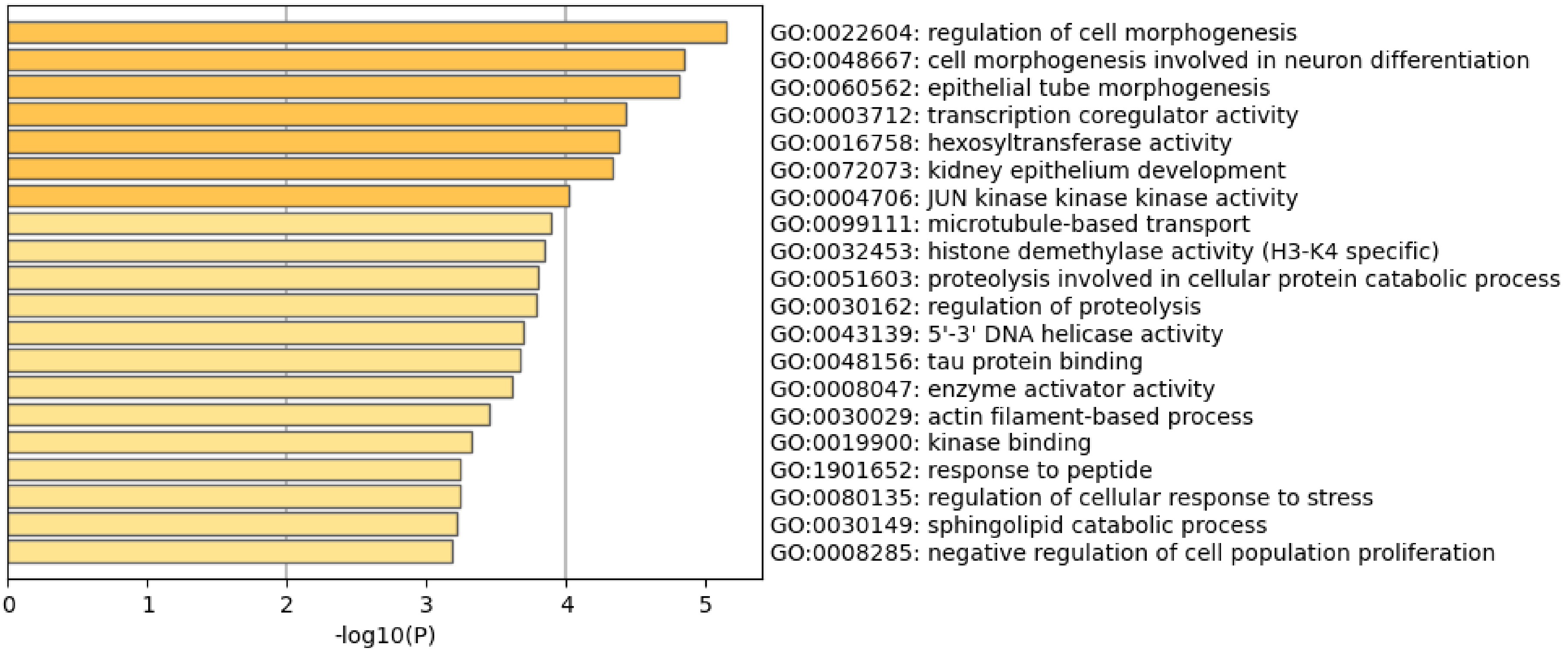

Supplementary Figure 6. Enrichment analysis with subtype-specific genes from METexp in IntSub2.

Reference

1. Robertson, A.G., et al., *Integrative Analysis Identifies Four Molecular and Clinical Subsets in Uveal Melanoma.* Cancer Cell, 2017. **32**(2): p. 204-220.e15.
